# Comparison and Evaluation of Outcomes of Ureteroscopy and Stone Laser Fragmentation in Extremes of Age Groups (≤10 Years and ≥80 Years of Age): A Retrospective Comparative Analysis of over 15 Years from 2 Tertiary European Centres

**DOI:** 10.3390/jcm12041671

**Published:** 2023-02-20

**Authors:** Mriganka M. Sinha, Amelia Pietropaolo, Yesica Quiroz Madarriaga, Erika Llorens de Knecht, Anna Bujons Tur, Stephen Griffin, Bhaskar K. Somani

**Affiliations:** 1 Urology Department, University Hospital Southampton NHS Trust, Southampton SO16 6YD, UK; 2 Pediatric Urology Unit, Urology Department, Fundació Puigvert, 08025 Barcelona, Spain; 3 Southampton Children Hospital NHS Trust, Southampton SO16 6YD, UK

**Keywords:** ureteroscopy, laser, kidney calculi, paediatric, urolithiasis, stent, elderly

## Abstract

Aim: To assess and compare the outcomes associated with ureteroscopy and laser fragmentation (URSL) for extremes of age group (≤10 and ≥80 years). Methods: Retrospective consecutive data were collected from two European centres for all paediatric patients ≤10 undergoing URSL over a 15-year period (group 1). It was compared to consecutive data for all patients ≥80 years (group 2). Data were collected for patient demographics, stone characteristics, operative details, and clinical outcomes. Results: A total of 168 patients had 201 URSL procedures during this time (74 and 94 patients in groups 1 and 2 respectively). The mean age and stone sizes were 6.1 years and 85 years, and 9.7 mm and 13 mm for groups 1 and 2 respectively. While the SFR was slightly higher in group 2 (92.5% versus 87.8%, *p* = 0.301), post-operative stent rate was also significantly higher in the geriatric population (75.9% versus 41.2%, *p* = 0.0001). There was also no significant difference in pre-operative stenting (*p* = 0.886), ureteric access sheath use (UAS) (*p* = 0.220) and post-operative complications. Group 1 had an intervention rate of 1.3/patient as compared to 1.1/patient in group 2. The overall complications were 7.2% and 15.3% in groups 1 and 2 respectively (0.069), with 1 Clavien IV complication related to post-operative sepsis and brief ICU admission in group 2. Conclusion: The paediatric population had a marginally higher incidence of repeat procedure, but the overall SFR and complications were similar, and post-operative stent insertion rates were much better compared to geriatric patients. URSL is a safe procedure in the extremes of age groups with no difference in the overall outcomes between the two groups.

## 1. Introduction

The global trend in lifetime prevalence of kidney stone disease (KSD) has increased from 10% to 14% in the last two decades [1,2,3]. The European Association of Urology (EAU) [4] in their 2021 guidelines recommend flexible ureteroscopy and lasertripsy (FURSL) as the first line of treatment in adults for uncomplicated ureteric and renal stones measuring less than 2 cm.

Surgical interventions such as FURSL can be associated with possible side effects and complications that are greatly dependent on patient age and comorbidities. Paediatric patients with renal tract calculi commonly have congenital anatomical abnormality and/or recurrent UTI and often benefit from smaller instruments. In recent years, the incidence of KSD in the paediatric population appears to be increasing [2]. Advances in ureteroscopic technology have allowed ureteroscopy to be adapted to the paediatric population [5]. Miniaturised ureteroscopes with sizes as small as 4.5 Fr have been created for paediatric cases to help improve the surgical outcome with minimal ureteral trauma [6]. These replace the historical 8.5/9.5 and 11 Fr scopes used in adults [7] The existing evidence for paediatric ureteroscopy for stone disease has demonstrated stone free rates (SFR) ranging between 58 and 100% [8,9] and low risk of complications; mainly Clavien–Dindo grade I–II [8].

Age progression towards geriatric population brings with it an entire cohort of physiological, anatomical and molecular changes. These translate into elderly patients presenting with multiple co-morbidities, possibly with pre-existing urinary symptoms requiring longer length of stay and more susceptible to general anaesthetic-associated complications [10]. Due to the increasing prevalence of KSD and need for intervention in both paediatric and geriatric age groups, we conducted this comparative retrospective analysis for these cohorts, and assessed the outcomes of FURSL, to evaluate its safety and efficacy in these two specific patient groups in the extremes of age groups (≤10 years and ≥80 years of age).

## 2. Materials and Methods

Retrospective data for consecutive paediatric procedures for patients ≤10 years of age from two tertiary paediatric endourological European centres (University Hospital Southampton, United Kingdom and Fundació Puigvert hospital, Spain) operating independently of each other were collected and analysed. This study was registered locally as an audit in University Hospital Southampton (audit number 6901) and was approved by the ethics committee in Fundació Puigvert hospital wherein all parents were consented for participation in the study. This was compared with retrospectively collected data for consecutive geriatric patients (≥80 years old) from the UK adult tertiary Endourological centre. The study was registered locally as an audit (audit number 6901) at University Hospital Southampton. The Fundació Puigvert hospital CEIM approved this study and family consent was obtained for all patients included in the study. The study period for the paediatric patients was from 2006–2021 (15 years) and adult patients from 2012–2021 (10 years).

A total of 201 FURSLs (168 patients) were performed in this time duration on 74 paediatric patients (group 1) and 94 geriatric patients (group 2). Data including age, gender, co-morbidities, American Society of Anaesthetics grading (ASA), symptom at time of presentation, laterality, stone size, site and biochemistry, date of surgery, duration of surgery, use of ureteric access sheath (UAS), pre and post-operative stent insertion, intra-operative and post-operative complications, stone free status, length of stay and follow-up were recorded. An intra-operative finding of being endoscopically stone free with post-operative imaging of fragments <2 mm was considered stone free.

The procedures were performed by experienced endourologists in both centres, and the data were collected independently by members of team not involved in the original procedure. Procedural details have been discussed and extensively detailed in the past (11–13). The diagnosis of stones was made by ultrasound scan (USS) and/or plain KUB XR, and non-contrast CT (NCCT) for groups 1 and 2 respectively. A follow-up USS was carried out for group 1 and a combination of USS (radiolucent stone) or KUB XR (radiopaque stone) or rarely NCCT (equivocal scan or persisting symptoms) for group 2 within 3 months of FURSL. A 4.5–6 F (Richard Wolf) semirigid scope and a 6 F (Storz) scope were used for groups 1 and 2 respectively. A Storz Flex X2 flexible ureteroscope [Karl Storz Endoscopy Ltd., Berkshire, UK] was used for all patients and while a 9.5/11.5 F ureteral access sheath (UAS) was used for group 1, a combination of 9.5/11.5 F or 12/14 F UAS was used for group 2. A Holmium:YAG laser [100 W, 60 W or 20 W Lumenis] was used for fragmentation using a 272-micron laser fibre (Lumenis, Inc., Santa Clara, CA, USA). Use of intra-operative UAS and post-operative stent was surgeon-dependent, and extracted stones were sent for crystallographic analysis.

Data were analysed and compared between the groups in terms of stone free rate, UAS use, stent use, complications and need for re-intervention. The data were initially collected in excel sheet (Microsoft, Redmond, WA, USA) and then anonymised and analysed in SPSS (IBM SPSS version 27). *p*-value was determined using chi-square test in SPSS for the statistical significance and a *p*-value of <0.05 was considered significant. The medians, standard deviation, range and percentage were calculated using excel.

## 3. Results

A total of 74 paediatric patients (group 1) and 94 geriatric patients (group 2) underwent 201 FURSL procedures during the study duration. The mean age and mean stone size for groups 1 and 2 was 6.1 years (range: 0.8–10 years) and 9.7 mm (range: 3–30 mm), and 85 years (range: 80–94 years) and 13 mm (range: 4–48 mm) respectively. The male: female ratio in group 1 was 1.4:1 vs. 2.4:1 in group 2 (Table 1).

A UAS was used in 19.5% and 25.9% in groups 1 and 2 respectively. While the rate of pre- and post-operative stent rates were 42.2% and 41.2% in group 1, it was 43.2% and 75.9% in group 2. The SFR was found to be marginally better in group 2 with a SFR of 92.5% vs. 87.8% in group 1 (*p* = 0.3). There were three minor ureteric injury in group 1 which were all managed conservatively with a ureteric stent, with no recorded intra-operative complications in group 2. Marginal differences were noted in post-operative complications between group 1 (7.2%) and group 2 (15.3%) (*p* = 0.069). The complications for group 1 were all Clavien–Dindo I/II and ranged from UTI/sepsis (*n* = 4), haematuria (*n* = 1), urinary retention and catheterisation (*n* = 2). The complications for group 2 were UTI/sepsis (*n* = 12), haematuria (*n* = 1), urinary retention and catheterisation (*n* = 1), temporary acute kidney injury (*n* = 1) and sepsis needing ICU admission (*n* = 1). These findings are enlisted in Table 2. The operative time for group 1 was 83.9 ± 42.2 mins vs. 47.06 ± 25.7 mins in group 2, which reflects the way in which the operative time was calculated. While group 1 included anaesthetic time as well as procedural time, group 2 only included the procedural time. The most common stone biochemistry in group 1 was found to be struvite stones while in group 2 was calcium oxalate stones. Only 36 out of 74 patients in group 1 had stone analysis as opposed to 85 out of 94 in group 2, therefore no statistical analysis has been performed for the stone biochemistry.

The median length of stay (LOS) in group 1 was 1.5 days (range: 0–12 days) vs. 0 days (range: 0–61 days) in group 2. The number of interventions needed to achieve stone free status were 1.3 in group 1 and 1.1 in group 2. The patients were followed up in outpatient clinic with X-ray kidney-ureter-bladder (KUB), USSKUB or CTKUB.

## 4. Discussion

Our study demonstrates safety and efficacy of FURSL in patients at extremes of age groups. We found a SFR of 87.8% and 92.5% in group 1 and 2 respectively, which is comparable to the previously published data [9,10,11,12,13,14]. All ureteric injuries found in group 1 were minor and managed conservatively. While there was a difference in post-operative stent usage between the groups with higher usage in group 2, there was no significant difference between the SFR although group 1 had higher mean procedure/patient ratio to achieve this. Similarly, infection related complication was higher in group 2, which could potentially be a reflection of pre-operative lower urinary tract symptoms or incomplete bladder drainage, but this was not captured in our study.

In their study of 80 ureterorenoscopies published in 2014, Azili et al. [15] found a significant relationship between URS required in infancy and the need to convert to open surgery. However, with miniaturization of paediatric scopes, improved optics and technology, coupled with better training opportunities for operating surgeons, the need for invasive surgery can be further minimised. Somani et al. [16] have given useful insight into ways to improve surgical outcomes for paediatric URS including multi-disciplinary team approach for planning and management via a twin-surgeon technique and approach.

In their systematic review, Rob et al. [17] found an over-all complication rate for paediatric FURSL at 11.1%, with 31% Clavien–Dindo (CD) II and III complications. Conversely, our study showed an overall complication rate of 7.2% in group 1, with all Clavien I/II complications. We have not found an increase in complications in younger children undergoing FURSL for kidney stones when compared against existing literature or against geriatric population [17], thereby reflecting safety of using FURSL for paediatric urolithiasis.

From the surgical point of view, FURSL is a minimally invasive procedure ensuring safety for this delicate patient cohort and the same efficacy provided as in adult patients can now be offered to paediatric patients [18]. Paediatric FURSL outcome has become superior to Extra-corporeal Shockwave Lithotripsy (ESWL) over the years [19]. In 2005, Tan et al. [20], mentioned the superiority of FURSL describing it as a first line option in stone treatment within the paediatric population. In a small patient group of 25 patients, they achieved a 95% SFR. These findings were confirmed by Thomas et al. [21]. More recently, Esposito et al. [22], compared the outcomes of FURSL between five paediatric high-volume centres finding a SFR of 97% and complication rates of 4%. Elsheemy et al. [23] in 2014 also analysed the outcome of 128 paediatric patients who underwent FURSL identifying that younger age and larger stones were predicting factors for post operative complications. However, a recent systematic review [17] underlined the importance of high volume experienced centres as a requirement for this type of specialist surgery and predictor of success.

At the other extreme of age, the geriatric population have different issues. These are often associated with general anaesthesia and possibly age related physiological and cognitive decline. The challenges due to age progression range from cardiovascular changes, presenting as higher blood pressures and reduced cardiac outputs, to decreased respiratory reserves due to suboptimal gas exchange along with reduced creatinine clearance and glomerular filtration causing renal dysfunction and poor drug elimination [24]. All the above, combined with increased susceptibility to post-operative confusion and delirium, refs. [24,25] require careful consideration and monitoring for GA administration in the geriatric population. Our study group had anaesthetic work up in preparation for surgery and careful consideration of anaesthetic and/or surgical needs in order to tailor them to the patients accordingly. Our median length of stay was 1.5 days in group 1 and 0 days in group 2 respectively, suggesting a quick recovery time in both these groups.

The definition of geriatric population in the literature is unclear [26] and ranged from over 65 years to over 75 years of age. Heyland et al. [27] in their prospective study of recovery after critical illness analysed 610 patients >80 years of age and found a significantly better outcome associated with younger age, lower frailty index and lower Charlson comorbidity score. They recommended assessment of frailty status and baseline physical function to improve outcomes in the elderly. In our study we found a 15.3% complication rate in the elderly with only one ICU admission for post-operative sepsis management. This is comparable to the existing evidence with 9% over-all complication rate found in patients >70 years of age by Prattley et al. [28] in their literature review of ureteroscopy for renal stone disease treatment. Emiliani et al. [29] compared the outcome of FURSL in both patients older and younger than 80 years old in a retrospective study. They found that despite the higher rate of comorbidity in the >80 patients’ group, the SFR and complication rate were similar, but the operative time and hospital stay were higher. It is recognised that elderly patients can more likely be affected by multiple comorbidities that often require them to be on long-term antiplatelet or anticoagulation medications. A multicentric study involving 31 centres and 9982 patients found that the risk of post operative hospitalization is increased in those taking antiplatelet therapy [30].

Berardinelli et al. [31] also analysed patients of different age groups defining elderly patient above 65 years of age. They found that despite showing a high Charlson comorbidity index compared to their younger counterpart, SFR, operative time and re-intervention rate did not show differences between the two groups. Equally, surgical and medical complication rates were similar between the two cohorts. Similar to our study, Tolga-Gulpinar et al. [13] subdivided their patients undergoing FURSL into multiple age groups. They found that overall complication rates in children were not statistically higher than in adult patients. Perioperative complications were not related to the age groups. Cakici et al. [32] also described elderly patients as above 60 years old and compared FURSL outcome between them and younger patients without identifying significant findings. Technological advances have now made ureteroscopy a frontline treatment for patients with stone disease in high risk patients including those at extremes of age [33,34,35]. A large multicentric global study from eight centres show that while ureteroscopy is acceptable as a first-line intervention in paediatric population, complications are higher in patients <5 years of age [36]. While group 1 included anaesthetic time as well as procedural time, group 2 only included the procedural time.

### Strengths, Limitations and Areas of Future Research

While our study includes consecutive patients for both groups, data analysis was retrospective in nature. Record keeping for procedural duration differed in the two groups in our study. While group 1 included anaesthetic time as well as procedural time, group 2 only included the procedural time. We therefore recommend that future studies standardise how procedural time is calculated. While the study includes patients from 2 centres, future prospective studies with more high-volume centres could lead to a more accurate comparison of outcome in various age groups and with different comorbidities. The stone free definition should also be standardised with more work focussed on both cost and quality of life [37,38]. A recent study has also recommended a paediatric ureteroscopy (P-URS) reporting checklist and nomogram to aid studies in how outcomes are reported [39,40].

## 5. Conclusions

In this study we found FURSL to be safe and effective for stone disease management with comparable SFR in both paediatric and geriatric cohorts despite the slightly higher rate of re-intervention in the paediatric age group. There was no significant difference in the use of UAS although significantly fewer paediatric patients were deemed to require a post-operative ureteral stent. The outcomes of our study show extremely favourable results of FURSL in extremes of age groups, and hence should be considered as a first line treatment for these patients.

## Figures and Tables

**Table 1:** Patient demographics in both patient cohorts undergoing FURSL (PUJ—pelvi-ureteric junction, VUJ—vesico-ureteric junction, NOS—not otherwise specified, ICU—intensive care unit and CD—Clavien–Dindo complication grade).

Demographics	Paediatric Group (≤10 Years)	Geriatric Group (≥80 Years)
Number of patients	74	94
Number of procedures	97	104
Procedure: patient	1.3:1	1.1:1
Mean age +/−SD (Range)	6.1 ± 2.4 (Range: 0.8–10 years)	85 ± 3.9 (Range: 80–94 years)
Male: Female	1.4:1	2.4:1
Mean stone size +/–SD (Range) in mm	9.7 ± 4.4 (3–30)	13± 8.2 (4–48)
**Stone location-Ureteric: Renal** **(Multiple stones)**	1:1.2 (35)	1.2:1 (25)
Renal pelvis	23	7
Upper renal pole	13	7
Middle renal pole	18	15
Lower renal pole	35	19
Proximal ureter/PUJ	4	11
Mid ureter	3	15
Distal ureter/VUJ	25	28
NOS	11	15
**Stone Biochemistry**		
Calcium Oxalate	2	34
Struvite	4	2
Calcium Phosphate	3	0
Cystine	3	0
Uric acid	0	2
Mixed biochemistry	1	37
Unspecified	23	10

**Table 2:** Intra-operative and post-operative outcomes of FURSL for both groups (CD—Clavien–Dindo classification of post-operative complications, UTI—urinary tract infection, ICU—intensive care unit, and AKI—acute kidney injury).

Details Compared	Paediatric Group (≤10 Years)	Geriatric Group (≥80 Years)	*p* Value
Duration of Surgery +/−SD	83.9 ± 42.2 mins	47.06 ± 25.7 mins	
Ureteric access sheath (UAS) use	19 (19.5%)	27 (25.9%)	0.220
Pre-operative stent	41/97 (42.2%)	45/104 (43.2%)	0.886
Post-operative stent	40/97 (41.2%)	79/104 (75.9%)	0.000
Stone free rate	87.8%	92.5%	0.301
Mean length of stay +/−SD (Range) in days	1.5 ± 1.7 (1–12)	0 ± 7.1 (0–61)	
Number of interventions/patient	1.3:1	1.1:1	
**Complications**			
*Intra-operative*			
Ureteric injury (stent inserted)	3	0	0.071
*Post-operative*			
Overall post-operative complications	7/97 (7.2%)	16/104 (15.3%)	0.069
Haematuria (CD)	1 (I)	1 (I)	0.960
UTI/sepsis	4 (II)	12 (II)	0.052
Sepsis requiring ICU admission	0	1 (IV)	0.333
Urine retention requiring catheterisation	2 (I)	1 (I)	0.520
Temporary AKI	0	1 (I)	0.333

## Data Availability

As data are identifiable, they will not be made available as per ethical approval.
